# *Chemistry Central Journal* themed issue: Dial-a-Molecule

**DOI:** 10.1186/s13065-015-0122-3

**Published:** 2015-08-16

**Authors:** Kelly J Kilpin, Richard J Whitby

**Affiliations:** Chemistry, Faculty of Natural and Environmental Sciences, University of Southampton, Southampton, SO17 1BJ UK

Chemical synthesis has taken huge steps forward over the last 30 years. Many new reactions have been added to the synthetic chemists toolbox, and old favorites have been studied in depth to increase both the scope and utility. Many complex molecules have been successfully synthesised for the first time, and for others dramatic improvements in route have been achieved. However, despite these synthetic developments, little has changed in the way synthesis is generally planned and carried out, which is somewhat surprising given the leaps forward both the technology and computing sectors have taken over the same time period.

Making molecules is not a trivial task—undergraduates through to experienced synthetic chemists will readily admit that the synthesis of somewhat modest targets requires a large investment of both time and effort. As an example, to achieve the total synthesis of a moderately complex molecule it may require 100 times more reactions than appear in the final route, largely due to reactions not working as expected and requiring development, or a change in the route.

At its inception, Dial-a-Molecule recognised these issues, and identified that if they were to be overcome synthetic chemistry must become a more data-driven discipline. It also needs to make far more use of automated equipment, particularly to improve the repeatability of, and information capture from, reactions. In order to realise the vision that “*in 20*–*40* *years, scientists will be able to deliver any desired molecule within a timeframe useful to the end*-*user, using safe, economically viable and sustainable processes*” synthetic chemistry must embrace the computing revolution that has taken place over the last 30 years, and take full advantage of the skill sets of researchers in areas outside of traditional chemistry disciplines.

To fully appreciate the difficulty of achieving the aims of Dial-a-Molecule, and the impact it will have once the vision is realised, it is useful to make a comparison with oligonucleotide synthesis. Automation of this process has hugely revolutionised molecular biology. The ability to quickly ‘dial-up’ any nucleotide sequence is now routine, but to get to this stage took 20 years of development. When you consider that these machines work with well-defined, structurally similar building blocks, using the same chemistry to link them, the enormity of the Grand Challenge becomes clear.

The benefits of being able to ‘dial-up’ any molecule will be immense, and reach far beyond the traditional chemistry end-users (e.g. the pharma industry). Ultimately, many emerging sectors such as (but not limited to) sustainable new materials, next generation electronics, healthcare and forensics, will benefit.

## The roadmap for chemical synthesis in the 21st century

Working towards achieving the Grand Challenge, the Dial-a-Molecule Network developed the Roadmap for Chemical Synthesis in the 21st century. The document was published in 2012 following extensive consultation involving academic and industrial representatives from across the community. It states the three key challenges in removing synthesis as a constraint to the access of any given molecule and achieving 100% efficiency:

- The need to make synthesis predictable.
- Developing smart synthesis by design.
- Providing sustainable synthetic routes to answer the need for a sustainable future.

**Fig. 1:**
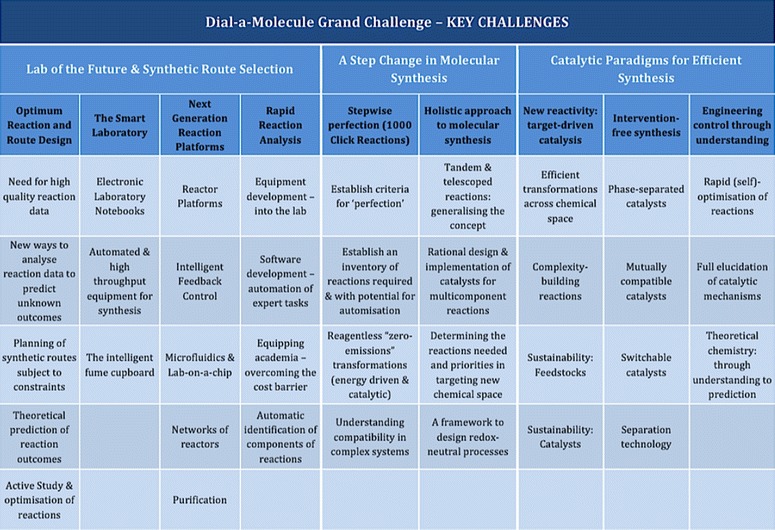
Key challenges identified in the Dial-a-Molecule Roadmap for Chemical Synthesis in the 21st century.

By breaking the Grand Challenge down into three inter-related themes (see Fig. 1 for a graphical representation, and below for a brief summary) the Roadmap defines the state of the art and identifies the gaps, opportunities and key actions to help guide research towards the realisation of the Dial-a-Molecule vision. The Roadmap is available to download at http://www.dial-a-molecule.org/roadmap.

### Lab of the future and synthetic route selection

Although the number, power and scope of synthetic reactions has increased significantly in recent times, the time and effort required to synthesise molecules has not. Retrosynthetic methods remain largely unchanged since they were introduced over 40 years ago, and in the majority of academic research labs synthetic procedures are still carried out manually. The Lab of the Future and Synthetic Route Selection theme describes how synthesis could be performed and the prediction of which synthetic routes to use. This theme is subdivided into four areas:

- *Optimum reaction and route design* describes how the optimum synthetic routes can be selected the first time by making use of data to predict the outcome of unknown reactions.
- The *smart laboratory* describes how reaction and process data can be acquired and shared between labs as a standard (and low cost) part of the synthetic workflow.
- *Next generation reaction platforms* redefines the technology used in chemical synthesis labs and describes the changes needed to make this technology widely accessible.
- *Rapid reaction analysis* defines the challenges of collecting and analysing full analytical data on reactions.

### A step change in molecule synthesis

Given unlimited time and resources the synthesis of most molecules could be achieved. Obviously this is not an acceptable or realistic scenario—funding measures and pressures from higher up the management chain all contribute towards the desire to synthesise molecules in a timely manner. Taking these factors into account, two approaches were identified to gain access to any molecule at will:

- *Stepwise perfection* (*1,000 click reactions*) If the synthetic chemist has access to enough perfect and reliable reactions, most complex molecules could be obtained in a step-wise, ‘clickable’ manner.
- The *holistic approach* defines the most direct way of moving from starting material to product by using a small number of reactions to generate complexity.

### Catalytic paradigms for efficient synthesis

Catalysis already plays a significant role in chemical synthesis and will continue to do so. It is undeniable that advances in bio-, metallo- and organocatalysis have had a significant and positive impact on synthesis—in the past 15 years two chemistry nobel prizes have been awarded for developments of catalytic methods. Regardless of these advances, challenges still remain and those central to achieving the Dial-a-Molecule vision can be subdivided into the following focus areas:

- *New reactivity, target*-*driven catalysis* looks at defining the catalysts and catalytic processes that will most advance the Dial-a-Molecule aims.
- *Intervention*-*free synthesis* is about sequencing catalytic reactions and minimising human intervention (increasing process efficiency).
- *Engineering control through understanding* is about using knowledge-based approaches to increase catalyst discovery and optimisation by combining experimental and theoretical approaches.

## The outlook

Grand challenges are defined as significant problems that need a long term, coordinated approach from researchers to overcome. Since it’s establishment in 2010, the Dial-a-Molecule community has grown to include almost 700 members, with one-third of these from non-academic institutes. Of the academics, almost half are not synthetic organic chemists. As a community, Dial-a-Molecule has run a number of meetings to discuss themes relevant to advancing the Roadmap, and through commissioned research projects has made significant inroads towards achieving many of the near and medium term goals. An extremely positive aspect is the community continues to grow, and the enthusiasm demonstrated by the next generation of researchers indicates that the research efforts towards achieving the aims of the Grand Challenge is in safe hands going forward.

This special edition in *Chemistry Central Journal* serves to highlight some of the recent efforts directed towards achieving the goals set out in the Roadmap.

